# (*E*)-5-[(3-Eth­oxy-2-hy­droxy­benzyl­idene)amino]-1,3,4-thia­diazole-2(3*H*)-thione

**DOI:** 10.1107/S1600536811049877

**Published:** 2011-11-25

**Authors:** Hadi Kargar, Reza Kia

**Affiliations:** a Department of Chemistry, Payame Noor University, PO BOX 19395-3697 Tehran, Iran; b X-ray Crystallography Lab., Plasma Physics Research Center, Science and Research Branch, Islamic Azad University, Tehran, Iran; c Department of Chemistry, Science and Research Branch, Islamic Azad University, Tehran, Iran

## Abstract

In the title compound, C_11_H_11_N_3_O_2_S_2_, the dihedral angle between the benzene ring and the five-membered ring is 6.85 (9)°. An intra­molecular O—H⋯N hydrogen bond makes an *S*(6) ring motif. In the crystal, mol­ecules are linked through bifurcated N—H⋯(O,O) hydrogen bonds with *R*_1_^2^(5) ring motifs, forming chains along the *b* axis. A short C⋯S contact [3.3189 (19) Å], which is shorter than the sum of the van der Waals radii of these atoms (3.50 Å), occurs in the structure. The crystal structure is further stabilized by C—H⋯N hydrogen bonding and π–π inter­actions [centroid–centroid distance = 3.7649 (12) Å].

## Related literature

For standard bond lengths, see: Allen *et al.* (1987 ▶). For hydrogen-bond motifs, see: Bernstein *et al.* (1995 ▶). For the biological versatility of thione ligands, see, for example: Kumar *et al.* (1988 ▶); Yadav *et al.* (1989 ▶). For related structures, see: Zhang (2003 ▶); Kargar *et al.*, (2011 ▶). For van der Waals radii, see: Bondi (1964 ▶).
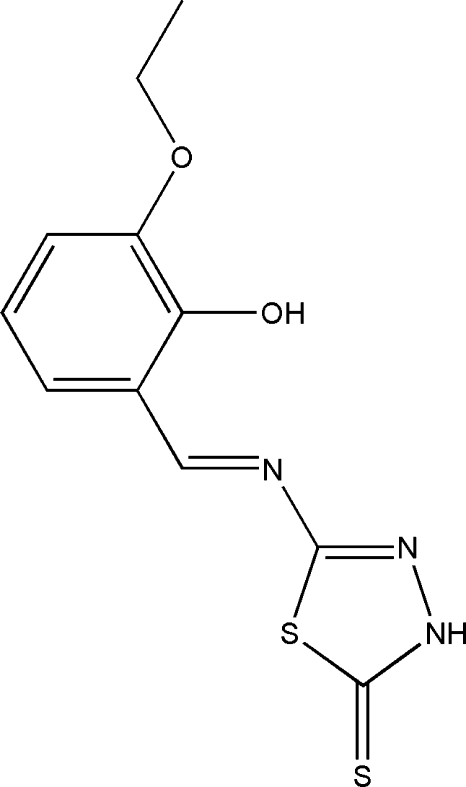


## Experimental

### 

#### Crystal data

C_11_H_11_N_3_O_2_S_2_
*M* *_r_* = 281.35
Monoclinic, 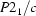

*a* = 8.925 (1) Å
*b* = 11.3664 (14) Å
*c* = 12.8945 (16) Å
β = 99.352 (9)°
*V* = 1290.7 (3) Å^3^
*Z* = 4
Mo *K*α radiation
μ = 0.41 mm^−1^
*T* = 291 K
0.25 × 0.22 × 0.15 mm

#### Data collection

Stoe IPDS 2T Image Plate diffractometer
Absorption correction: multi-scan [*MULABS* (Blessing, 1995 ▶) in *PLATON* (Spek, 2009 ▶)] *T*_min_ = 0.905, *T*_max_ = 0.941
10227 measured reflections
3461 independent reflections
2376 reflections with *I* > 2σ(*I*)
*R*_int_ = 0.036

#### Refinement

*R*[*F* ^2^ > 2σ(*F* ^2^)] = 0.046
*wR*(*F* ^2^) = 0.103
*S* = 1.00
3461 reflections
164 parameters
H-atom parameters constrained
Δρ_max_ = 0.24 e Å^−3^
Δρ_min_ = −0.30 e Å^−3^

### 

Data collection: *X-AREA* (Stoe & Cie, 2009 ▶); cell refinement: *X-AREA*; data reduction: *X-AREA*; program(s) used to solve structure: *SHELXTL* (Sheldrick, 2008 ▶); program(s) used to refine structure: *SHELXTL*; molecular graphics: *SHELXTL*; software used to prepare material for publication: *SHELXTL* and *PLATON* (Spek, 2009 ▶).

## Supplementary Material

Crystal structure: contains datablock(s) global, I. DOI: 10.1107/S1600536811049877/ff2044sup1.cif

Structure factors: contains datablock(s) I. DOI: 10.1107/S1600536811049877/ff2044Isup2.hkl

Supplementary material file. DOI: 10.1107/S1600536811049877/ff2044Isup3.cml

Additional supplementary materials:  crystallographic information; 3D view; checkCIF report

## Figures and Tables

**Table 1:** Hydrogen-bond geometry (Å, °)

*D*—H⋯*A*	*D*—H	H⋯*A*	*D*⋯*A*	*D*—H⋯*A*
O1—H1⋯N1	0.87	1.81	2.5924 (19)	150
N3—H3⋯O1^i^	0.83	2.15	2.841 (2)	141
N3—H3⋯O2^i^	0.83	2.47	3.160 (2)	142
C3—H3*A*⋯N2^ii^	0.93	2.60	3.312 (3)	133

Symmetry codes: (i) 
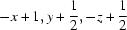
; (ii) 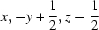.

**Fig. 1.**
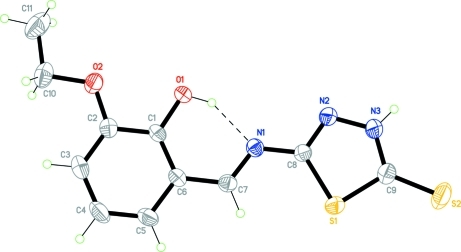
The ORTEP plot of the title compound, showing 40% probability displacement ellipsoids and the atomic numbering. The dashed lines show the intermolecular interaction.

**Fig. 2.**
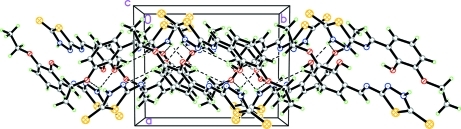
The packing diagram of the title compound viewed down the c-axis showing linkning of molecules through the intermolecular N—H···O hydrogen bonds, forming one-dimensional extended chains along the b-axis. The dashed lines show the intermolecular interactions.

## supplementary crystallographic information

### Comment

The biological versatility of compounds incorporating a thiadiazole ring is well known (Kumar *et al.*, 1988; Yadav *et al.*, 1989).

The asymmetric unit of the title compound, Fig. 1, comprises a thione-Schiff base ligand. The bond lengths (Allen *et al.*, 1987) and angles are within the normal ranges and are comparable to the related structures (Zhang, 2003; Kargar *et al.*, 2011).

The dihedral angle between the benzene ring and the five-membered ring is 6.85 (9)°. The intramolecular O—H···N hydrogen bond makes S_2_^2^(6) ring motif (Bernstein *et al.*,1995). In the crystal packing molecules are linked together through bifurcated N—H···O hydrogen bonds with R^2^_1_(5) ring motifs (Bernstein *et al.*,1995), forming one-dimensional extended chains along the *b* axis. The interesting feature of the crystal structure is the short C7···S2 contact [3.3189 (19)Å; (i) 2 - x, 1/2 + y, 1/2 - z], which is shorter than the sum of the van der Waals radii of these atoms [3.50Å]. The crystal structure is further stabilized by the intermolecular C—H···N hydrogen bonds and π-π interaction [*Cg1*···*Cg2*^i^ = 3.7649 (12)Å, (i) 1 - *x*, 1 - *y*, -*z*; *Cg1* and *Cg2* are centroids of S(1)/C(8)/N(2)/N(3)/C(9) and C1–C6 rings, respectively].

### Experimental

The title compound was synthesized by adding 3-ethoxy-salicylaldehyde (1 mmol) to a solution of 5-aminothiophene-2-thiol (1 mmol) in ethanol (30 ml). The mixture was refluxed with stirring for half an hour. The resultant solution was filtered. Yellow single crystals of the title compound suitable for *X*-ray structure determination were recrystallized from ethanol by slow evaporation of the solvents at room temperature over several days.

### Refinement

All hydrogen atoms were positioned geometrically with C—H = 0.93–0.97 Å and included in a riding model approximation with *U*_iso_ (H) = 1.2 or 1.5 *U*_eq_ (C). A rotating group model was applied to the methyl group.

### Crystal data

**Table:**

C_11_H_11_N_3_O_2_S_2_	*F*(000) = 584
*M**_r_* = 281.35	*D*_x_ = 1.448 Mg m^−^^3^
Monoclinic, *P*2_1_/*c*	Mo *K*α radiation, λ = 0.71073 Å
Hall symbol: -P 2ybc	Cell parameters from 8153 reflections
*a* = 8.925 (1) Å	θ = 1.8–29.6°
*b* = 11.3664 (14) Å	µ = 0.41 mm^−^^1^
*c* = 12.8945 (16) Å	*T* = 291 K
β = 99.352 (9)°	Block, yellow
*V* = 1290.7 (3) Å^3^	0.25 × 0.22 × 0.15 mm
*Z* = 4	

### Data collection

**Table:**

Stoe IPDS 2T Image Plate diffractometer	3461 independent reflections
Radiation source: fine-focus sealed tube	2376 reflections with *I* > 2σ(*I*)
graphite	*R*_int_ = 0.036
φ and ω scans	θ_max_ = 29.2°, θ_min_ = 2.3°
Absorption correction: multi-scan [*MULABS* (Blessing, 1995) in *PLATON* (Spek, 2009)]	*h* = −12→12
*T*_min_ = 0.905, *T*_max_ = 0.941	*k* = −15→15
10227 measured reflections	*l* = −15→17

### Refinement

**Table:**

Refinement on *F*^2^	Primary atom site location: structure-invariant direct methods
Least-squares matrix: full	Secondary atom site location: difference Fourier map
*R*[*F*^2^ > 2σ(*F*^2^)] = 0.046	Hydrogen site location: inferred from neighbouring sites
*wR*(*F*^2^) = 0.103	H-atom parameters constrained
*S* = 1.00	*w* = 1/[σ^2^(*F*_o_^2^) + (0.0529*P*)^2^] where *P* = (*F*_o_^2^ + 2*F*_c_^2^)/3
3461 reflections	(Δ/σ)_max_ = 0.001
164 parameters	Δρ_max_ = 0.24 e Å^−^^3^
0 restraints	Δρ_min_ = −0.30 e Å^−^^3^

### Special details

**Table:**

Geometry. All e.s.d.'s (except the e.s.d. in the dihedral angle between two l.s. planes) are estimated using the full covariance matrix. The cell e.s.d.'s are taken into account individually in the estimation of e.s.d.'s in distances, angles and torsion angles; correlations between e.s.d.'s in cell parameters are only used when they are defined by crystal symmetry. An approximate (isotropic) treatment of cell e.s.d.'s is used for estimating e.s.d.'s involving l.s. planes.
Refinement. Refinement of *F*^2^ against ALL reflections. The weighted *R*-factor *wR* and goodness of fit *S* are based on *F*^2^, conventional *R*-factors *R* are based on *F*, with *F* set to zero for negative *F*^2^. The threshold expression of *F*^2^ > σ(*F*^2^) is used only for calculating *R*-factors(gt) *etc*. and is not relevant to the choice of reflections for refinement. *R*-factors based on *F*^2^ are statistically about twice as large as those based on *F*, and *R*- factors based on ALL data will be even larger.

### Fractional atomic coordinates and isotropic or equivalent isotropic displacement parameters (Å^2^)

**Table:**

	*x*	*y*	*z*	*U*_iso_*/*U*_eq_	
S1	0.87646 (6)	0.66233 (4)	0.17887 (4)	0.04681 (15)	
S2	0.95905 (7)	0.83877 (5)	0.35481 (6)	0.0674 (2)	
O1	0.46721 (15)	0.31614 (11)	0.07089 (10)	0.0420 (3)	
H1	0.5138	0.3743	0.1056	0.063*	
O2	0.37731 (16)	0.13586 (11)	−0.04539 (11)	0.0464 (3)	
N1	0.66203 (16)	0.48422 (12)	0.12047 (11)	0.0343 (3)	
N2	0.65853 (18)	0.59227 (13)	0.27073 (13)	0.0431 (4)	
N3	0.73429 (19)	0.68024 (13)	0.32832 (13)	0.0459 (4)	
H3	0.7041	0.7048	0.3820	0.055*	
C1	0.54149 (19)	0.29557 (15)	−0.01085 (12)	0.0324 (4)	
C2	0.4937 (2)	0.19863 (15)	−0.07513 (14)	0.0364 (4)	
C3	0.5647 (3)	0.17501 (17)	−0.16015 (15)	0.0473 (5)	
H3A	0.5343	0.1104	−0.2026	0.057*	
C4	0.6810 (3)	0.2464 (2)	−0.18320 (17)	0.0564 (6)	
H4A	0.7274	0.2295	−0.2410	0.068*	
C5	0.7277 (2)	0.34171 (19)	−0.12110 (16)	0.0481 (5)	
H5A	0.8046	0.3900	−0.1376	0.058*	
C6	0.6600 (2)	0.36673 (15)	−0.03266 (13)	0.0348 (4)	
C7	0.7168 (2)	0.46283 (15)	0.03607 (14)	0.0355 (4)	
H7A	0.7943	0.5100	0.0190	0.043*	
C8	0.72069 (18)	0.57225 (14)	0.18892 (13)	0.0327 (4)	
C9	0.8533 (2)	0.73096 (16)	0.29585 (15)	0.0410 (4)	
C10	0.3216 (3)	0.03555 (18)	−0.10841 (18)	0.0571 (6)	
H10A	0.3994	−0.0246	−0.1040	0.069*	
H10B	0.2931	0.0585	−0.1814	0.069*	
C11	0.1864 (3)	−0.0103 (2)	−0.0663 (2)	0.0802 (8)	
H11A	0.1507	−0.0808	−0.1032	0.120*	
H11B	0.1073	0.0479	−0.0759	0.120*	
H11C	0.2143	−0.0273	0.0072	0.120*	

### Atomic displacement parameters (Å^2^)

**Table:**

	*U*^11^	*U*^22^	*U*^33^	*U*^12^	*U*^13^	*U*^23^
S1	0.0386 (3)	0.0513 (3)	0.0539 (3)	−0.0096 (2)	0.0173 (2)	−0.0106 (2)
S2	0.0545 (3)	0.0618 (4)	0.0836 (5)	−0.0179 (3)	0.0043 (3)	−0.0267 (3)
O1	0.0520 (8)	0.0428 (7)	0.0348 (7)	−0.0101 (6)	0.0177 (6)	−0.0064 (5)
O2	0.0550 (8)	0.0394 (7)	0.0444 (8)	−0.0097 (6)	0.0067 (6)	−0.0034 (6)
N1	0.0360 (8)	0.0322 (7)	0.0349 (8)	0.0024 (6)	0.0061 (6)	−0.0007 (6)
N2	0.0478 (9)	0.0386 (8)	0.0459 (9)	−0.0094 (7)	0.0163 (7)	−0.0090 (7)
N3	0.0511 (10)	0.0440 (8)	0.0460 (9)	−0.0089 (7)	0.0174 (8)	−0.0154 (7)
C1	0.0367 (9)	0.0349 (8)	0.0261 (8)	0.0069 (7)	0.0062 (7)	0.0013 (6)
C2	0.0406 (9)	0.0347 (8)	0.0322 (9)	0.0042 (7)	0.0011 (7)	0.0025 (7)
C3	0.0592 (12)	0.0446 (10)	0.0378 (10)	0.0063 (9)	0.0066 (9)	−0.0100 (8)
C4	0.0604 (13)	0.0683 (14)	0.0451 (12)	0.0048 (11)	0.0221 (10)	−0.0173 (10)
C5	0.0426 (10)	0.0616 (12)	0.0438 (11)	−0.0024 (9)	0.0182 (9)	−0.0077 (9)
C6	0.0349 (9)	0.0398 (9)	0.0301 (8)	0.0054 (7)	0.0065 (7)	−0.0012 (7)
C7	0.0310 (9)	0.0384 (9)	0.0372 (9)	0.0012 (7)	0.0062 (7)	0.0019 (7)
C8	0.0323 (8)	0.0297 (8)	0.0369 (9)	0.0020 (7)	0.0077 (7)	0.0006 (7)
C9	0.0359 (9)	0.0380 (9)	0.0478 (11)	0.0030 (8)	0.0031 (8)	−0.0062 (8)
C10	0.0692 (15)	0.0437 (11)	0.0527 (13)	−0.0090 (10)	−0.0070 (11)	−0.0053 (9)
C11	0.0754 (18)	0.0700 (16)	0.090 (2)	−0.0320 (14)	−0.0014 (15)	−0.0004 (14)

### Geometric parameters (Å, °)

**Table:**

S1—C9	1.7400 (19)	C2—C3	1.379 (3)
S1—C8	1.7483 (17)	C3—C4	1.387 (3)
S2—C9	1.6544 (19)	C3—H3A	0.9300
O1—C1	1.354 (2)	C4—C5	1.371 (3)
O1—H1	0.8659	C4—H4A	0.9300
O2—C2	1.365 (2)	C5—C6	1.404 (3)
O2—C10	1.441 (2)	C5—H5A	0.9300
N1—C7	1.287 (2)	C6—C7	1.446 (2)
N1—C8	1.380 (2)	C7—H7A	0.9300
N2—C8	1.290 (2)	C10—C11	1.495 (3)
N2—N3	1.358 (2)	C10—H10A	0.9700
N3—C9	1.335 (2)	C10—H10B	0.9700
N3—H3	0.8311	C11—H11A	0.9600
C1—C6	1.396 (2)	C11—H11B	0.9600
C1—C2	1.403 (2)	C11—H11C	0.9600
			
C9—S1—C8	89.45 (9)	C1—C6—C7	121.06 (15)
C1—O1—H1	106.2	C5—C6—C7	119.78 (17)
C2—O2—C10	117.63 (16)	N1—C7—C6	121.18 (16)
C7—N1—C8	121.41 (15)	N1—C7—H7A	119.4
C8—N2—N3	109.58 (15)	C6—C7—H7A	119.4
C9—N3—N2	119.82 (15)	N2—C8—N1	118.84 (15)
C9—N3—H3	119.9	N2—C8—S1	114.22 (13)
N2—N3—H3	120.2	N1—C8—S1	126.94 (13)
O1—C1—C6	122.67 (15)	N3—C9—S2	126.83 (15)
O1—C1—C2	117.08 (15)	N3—C9—S1	106.92 (13)
C6—C1—C2	120.25 (16)	S2—C9—S1	126.25 (12)
O2—C2—C3	126.19 (16)	O2—C10—C11	107.2 (2)
O2—C2—C1	114.63 (15)	O2—C10—H10A	110.3
C3—C2—C1	119.18 (17)	C11—C10—H10A	110.3
C2—C3—C4	120.87 (18)	O2—C10—H10B	110.3
C2—C3—H3A	119.6	C11—C10—H10B	110.3
C4—C3—H3A	119.6	H10A—C10—H10B	108.5
C5—C4—C3	120.29 (18)	C10—C11—H11A	109.5
C5—C4—H4A	119.9	C10—C11—H11B	109.5
C3—C4—H4A	119.9	H11A—C11—H11B	109.5
C4—C5—C6	120.27 (19)	C10—C11—H11C	109.5
C4—C5—H5A	119.9	H11A—C11—H11C	109.5
C6—C5—H5A	119.9	H11B—C11—H11C	109.5
C1—C6—C5	119.12 (16)		
			
C8—N2—N3—C9	0.1 (3)	C4—C5—C6—C7	175.99 (19)
C10—O2—C2—C3	−0.4 (3)	C8—N1—C7—C6	176.85 (15)
C10—O2—C2—C1	179.53 (16)	C1—C6—C7—N1	1.4 (3)
O1—C1—C2—O2	−0.7 (2)	C5—C6—C7—N1	−176.53 (17)
C6—C1—C2—O2	179.79 (15)	N3—N2—C8—N1	178.96 (15)
O1—C1—C2—C3	179.21 (16)	N3—N2—C8—S1	−0.3 (2)
C6—C1—C2—C3	−0.3 (3)	C7—N1—C8—N2	177.93 (17)
O2—C2—C3—C4	179.21 (19)	C7—N1—C8—S1	−2.9 (2)
C1—C2—C3—C4	−0.7 (3)	C9—S1—C8—N2	0.27 (15)
C2—C3—C4—C5	0.3 (3)	C9—S1—C8—N1	−178.89 (16)
C3—C4—C5—C6	1.0 (3)	N2—N3—C9—S2	179.90 (14)
O1—C1—C6—C5	−177.84 (16)	N2—N3—C9—S1	0.1 (2)
C2—C1—C6—C5	1.6 (3)	C8—S1—C9—N3	−0.18 (14)
O1—C1—C6—C7	4.2 (3)	C8—S1—C9—S2	179.99 (14)
C2—C1—C6—C7	−176.35 (15)	C2—O2—C10—C11	−174.57 (18)
C4—C5—C6—C1	−2.0 (3)		

### Hydrogen-bond geometry (Å, °)

**Table:**

*D*—H···*A*	*D*—H	H···*A*	*D*···*A*	*D*—H···*A*
O1—H1···N1	0.87	1.81	2.5924 (19)	150
N3—H3···O1^i^	0.83	2.15	2.841 (2)	141
N3—H3···O2^i^	0.83	2.47	3.160 (2)	142
C3—H3A···N2^ii^	0.93	2.60	3.312 (3)	133

Symmetry codes: (i) −*x*+1, *y*+1/2, −*z*+1/2; (ii) *x*, −*y*+1/2, *z*−1/2.
